# Editorial: Disaster-related psychiatric disorders: assessment, recovery, intervention

**DOI:** 10.3389/fpsyt.2025.1586458

**Published:** 2025-03-25

**Authors:** Samuel F. Law

**Affiliations:** Department of Psychiatry, Faculty of Medicine, University of Toronto, Toronto, ON, Canada

**Keywords:** PTSD, resilience, disaster, trauma, public health

Studies of the 9/11 attack in the US have led to new critical understanding and insights into disaster psychiatry, highlighting screening, trauma responses, long-term mental health impacts, and intervention strategies. Related research have paved the way to increased public awareness of Post-Traumatic Distress Disorder (PTSD) and their impact on survivors, first responders, and families (1). There have also been renewed focus on early intervention efforts, and mindfulness that PTSD often exacerbate previous vulnerabilities and typically coexists with other mental illness such as depression, anxiety, and substance use disorders (2), calling for integrated treatment approaches (3). It has also pointed to long-term effects of PTSD, affecting some survivors with persistent mental health challenges, and how symptoms sometimes worsen over time (4). The field has learned to shift towards more community-based interventions, resilience training, and development of more mental health programs tailored to trauma care (5).

In this Research Topic: *Disaster-Related Psychiatric Disorders: Assessment, Recovery, Intervention*, researchers have continued to expand our knowledge in disaster and trauma related effects, studying numerous man-made and natural disasters, with Covid-19 being a global threat as another important backdrop. While the current understanding is that disasters and their impacts are highly heterogeneous (6), these studies contribute nuanced new knowledge that are informative to the field at large.

In Lee et al. studies of bereaved families from a fairy disaster in Korea, they showed long-term prevalence of PTSD symptoms in family members who lost loved ones, and highlighted how psychological state of optimism are protective against PTSD, but being avoidant and isolative worsened such. The presence of these factors likely reflect both personal and cultural attributes, providing potential area of foci in treatment and support. Addressing another disaster, studies by Berthail et al. on the Paris attack in 2015 drew newer attention to the fact that physical reactions to the traumatic event and tendency to engage in intrusive thoughts suppression are associated with elevated development of PTSD. These findings call for attention to both psychological and biological underpinnings for PTSD, and could contribute to early screening success for those who will be vulnerable in developing PTSD. To focus on protective factors, First’s study focused on disaster resilience, conceptualized as how various internal and external factors interact to influence an individual’s ability to adapt and recover following exposure to a disaster. The knowledge that such factors related to resilience can modify development of PTSD and depression give rise to areas of interest for both population and clinical levels of intervention. The study also found that more exposure to disaster losses was associated with more resilience, so exposure to disaster is related to development of resilience itself, as intuitive as it may sound; however, there is some critical doses of such loss that can overwhelm an individual’s resilience when the exposure levels are cumulative and ongoing (7), with an implication that timely and targeted support is critical in fostering resilience. Moving the field more upstream and using a preventative framework, Rizzi et al. have studied earthquake survivors in Morocco, particularly in a highly vulnerable population of pediatric cancer patients and caregivers, and advocated a focus on the communication, education, and having well defined disaster preparedness plans as a way to increase population health and resilience.

In a parallel world to the local and single event disasters, the Covid pandemic with its protracted and global impact at social, economical, and population health levels has trained a spotlight on the importance of disaster preparation and management. Out of this global disaster, a number of authors have contributed work to further our understanding on the manifested impact the pandemic has had. Yuan et al. have reported how the pandemic disaster could exacerbate a previously vulnerable and disadvantaged population living with HIV in a large Chinese sample of participants. Medved et al. remarked on increase in substance use and addictive behaviors in those with severe mental illness, occurring during the pandemic and compounded by the disaster of an earthquake in Croatia. The double-disaster predicted the acute need for special attention for those who are already vulnerable. Combined, these authors point to an importance of a more progressive, selective approach in protecting the most vulnerable during challenging times (8).

In other fronts, Teckchandani et al. examined those who were not known yet to be vulnerable, but found those with higher potential to have mental health concerns tend to be less likely to engage in some forms of proactive mental health training in a police cadet population that is known to face higher incidences of mental health challenge, including PTSD as part of their professional hazard. Their study is noteworthy for how to identify and engage in an upstream approach for health promotion and illness prevention. Another vulnerable group, by choice or by external factors is people with a history of migration. Renner et al. reviewed the relationship between migratory grief –defined as related to interpersonal, material and abstract losses - and psychopathology. Not surprisingly, migratory grief predicted depression and psychological distress, but their public health and social policy implications are ever more relevant in today’s global political climate regarding immigration and resettlement issues. Lastly, taking on actual studies of refugee youth, Schumacher et al. examined PTSD and beyond in refugee minors in Germany, and found a large proportion over (40%) of the participants met diagnostic criteria for depression and many of whom showed comorbid PTSD diagnosis. They stressed the importance of recognizing latent depressive symptoms that develop from the original PTSD symptoms associated with the flight and stress as part of the refugee experience for many.

In summary, the Research Topic in disaster related psychiatric disorders expanded knowledge in assessment, understanding, and paved new paths towards more effective and tailored intervention, with aim to promote fuller recovery, particularly mindful of those who are already or known to be vulnerable. Studying these populations from the margins could ultimately help us to understand more on how to address disaster related mental health challenges for the general population at large.
